# Comparison of Micro-Percutaneous and Mini-Percutaneous Nephrolithotomy in the Treatment of Renal Stones: A Systematic Review and Meta-Analysis

**DOI:** 10.3389/fsurg.2021.743017

**Published:** 2021-10-04

**Authors:** Xiaoshuai Gao, Wei Wang, Liao Peng, Xingpeng Di, Kaiwen Xiao, Jixiang Chen, Tao Jin

**Affiliations:** Department of Urology, Institute of Urology (Laboratory of Reconstructive Urology), West China Hospital, Sichuan University, Chengdu, China

**Keywords:** micro-percutaneous nephrolithotomy, mini-percutaneous nephrolithotomy, kidney stones, stone-free rate, meta-analysis

## Abstract

**Background:** To assess the efficacy and safety of micro-percutaneous nephrolithotomy (Microperc) and mini-percutaneous nephrolithotomy (Miniperc) in the treatment of moderately sized renal stones.

**Methods:** Literature search of PubMed, Web of Science, and Embase was performed prior to January 2021. We used odds ratios (OR) and weighted mean difference (WMD) for dichotomous variables and continuous variables, respectively. Results were pooled using Review Manager version 5.3 software.

**Results:** A total of six studies involving 291 Microperc and 328 Miniperc cases was included. The overall stone-free rate (SFR) of Microperc was 87.29% (254/291), while the SFR of Miniperc was 86.59% (284/328). Microperc was associated with lower hemoglobin drop (WMD: −0.98; *P* = 0.03) and higher renal colic requiring D-J stent insertion (OR: 3.49; *P* = 0.01). No significant differences existed between Microperc and Miniperc with respect to SFR (OR: 1.10; *P* = 0.69), urinary tract infection (OR: 0.38; *P* = 0.18), operative time (WMD: −5.76; *P* = 0.62), and hospital stay time (WMD: −1.04; *P* = 0.07).

**Conclusions:** Our meta-analysis demonstrated that Microperc could produce an SFR that was comparable with that of Miniperc. Microperc was associated with lower hemoglobin drop, while Miniperc was associated with lower renal colic rates. In addition, the operation time and hospital stay time for both these procedures were similar.

## Introduction

Kidney stone disease has affected humankind since antiquity, and its incidence has increased to almost 9% during the recent decades (1). Moreover, studies have shown that the risks associated with the recurrence of kidney stones can be as high as 50% (2). Current minimally invasive treatment procedures for kidney stones include extracorporeal shock wave lithotripsy (ESWL), retrograde intrarenal surgery (RIRS), and percutaneous nephrolithotomy (PCNL) (3). PCNL has the advantage of a high stone-free rate (SFR), but its higher efficiency is accompanied by more complications (4).

To reduce the complications resulting from PCNL, Miniperc (10–20 F) and Microperc (4.85 F) are now being implemented (5). Miniperc, Microperc, RIRS, and ESWL are the main surgical procedures for treating moderately sized renal stones. Although ESWL is recommended for renal stones of size <2 cm, its SFR is lower than that of other surgical methods, especially for lower pole stones (6, 7). The previous meta-analysis demonstrated that the SFR of Miniperc was higher than that of RIRS, and the overall complications were similar for both the procedures (8). Another meta-analysis demonstrated that Microperc was also associated with a higher SFR compared to RIRS (9). Both Miniperc and Microperc are efficient surgeries for treating renal stones. However, there are a few studies that directly assess the efficacy of Miniperc and Microperc, and hence a controversy still exists regarding which surgery is more advantageous. Therefore, we hope to review the previous literature, pool the data, systematically evaluate the advantages and disadvantages of Miniperc and Microperc, and provide convincing guidance for the clinical treatment of moderately sized renal stones.

## Materials and Methods

### Search Strategy

Literature search of PubMed, Web of Science, and Embase was performed prior to January 2021 according to the PRISMA guidelines (10). The following terms were used for the search: “micro-percutaneous nephrolithotomy,” “micro-PCNL,” or “micro-perc;” “Minipercutaneous,” “mini-PCNL,” “ultra-mini-PCNL,” “minimally invasive PCNL,” “minimal tract,” “miniaturized PCNL,” “miniperc,” or “MPCNL;” and “renal stone” or “calculi”.

### Inclusion Criteria and Exclusion Criteria

All eligible researches were selected based on the following criteria: (1) patients with renal stones of size < 2.5 cm, (2) experimental studies comparing Microperc with Miniperc, and (3) studies that reported at least one of the following items: mean operative time, SFR, hospitalization time, and complications (renal colic requiring D-J stent insertion, hemoglobin drop, and urinary tract infection). Exclusion criteria included (1) non-English papers, (2) conference abstract, (3) non-comparative studies (letters, comments, and reviews), and (4) those not included in the inclusion criteria.

### Quality Assessment

Two authors (XSG and WW) evaluated the literature quality independently, and the differences, if any, were discussed with the third author and solved. We assessed the level of evidence (LE) according to the Oxford Center for Evidence-based Medicine (11). We assessed the methodological quality of the included studies on the basis of the Newcastle Ottawa Scale (NOS) (12).

### Data Extraction

We read the full text of the included articles and extracted details regarding the author, publication date, study type, access sheath size of Miniperc, inclusion criteria, follow-up imaging and duration, number of patients, gender, age, stone side, stone size, operation time, stone-free rate, hospital stay, and complications (D-J stent insertion, hemoglobin drop, and urinary tract infection).

### Statistical Analysis

All meta-analyses were performed using Review Manager version 5.3 software. Odds ratios (OR) and 95% confidence interval (CI) were used for the dichotomous variables, while weighted mean difference (WMD) and 95% CI were used for the continuous variables. We assessed the heterogeneity of the included studies by Cochrane Chi-square test and *I*^2^ test. Fixed-effects model was used for low heterogeneity among studies (*P* > 0.1, *I*^2^ < 50%); otherwise, random-effects model was used when there was evidence of high heterogeneity (*P* < 0.1, *I*^2^ > 50%). Pooled effects were calculated using *Z*-test, and *P* < 0.05 was considered statistically significant. Sensitivity analysis was conducted using a single-item removal method. The funnel plot was used to assess the potential publication bias.

## Results

### Characteristics of Studies and Quality Assessment

**Figure 1:**
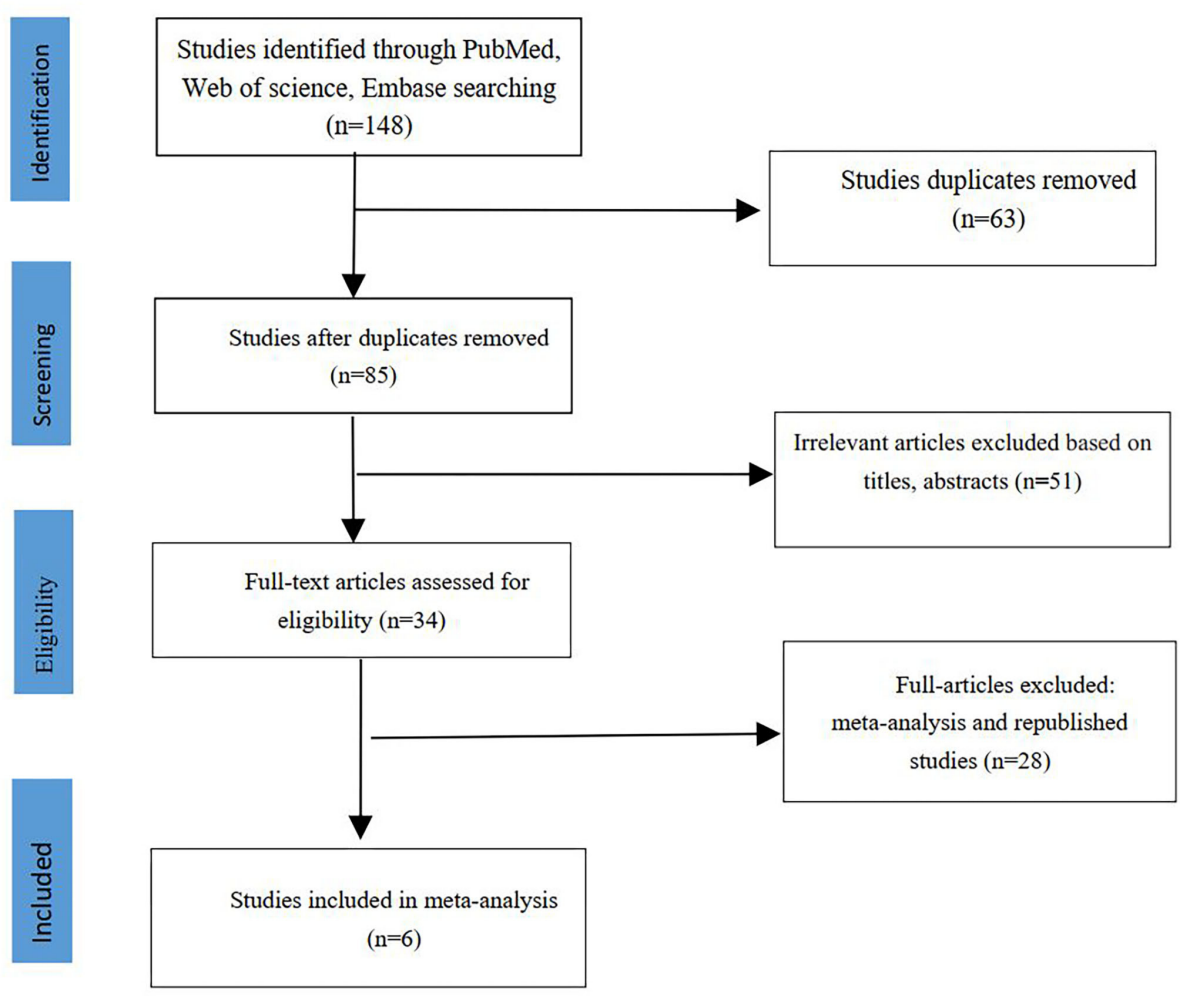
The flowchart showing study search and selection process.

The literature search protocol is presented in Figure 1. Following our search strategy, we initially retrieved 148 articles. Finally, six studies including 291 Microperc and 328 Miniperc cases were included in our meta-analysis (13–18). All the six studies that were included were retrospective case-control trials. These studies compared the advantages and disadvantages of the two surgical methods for treating moderately sized kidney stones. These six studies included three studies involving adult kidney stones (13, 14, 16) and three studies involving pediatric kidney stones (15, 17, 18). The LE of the six included studies was 3b, and the NOS score of these studies ranged from 6 to 7. The characteristics of these studies are listed in Table 1.

**Table 1:** Basic characteristics and data of included studies.

**Study**	**Study design**	**LE**	**Tract size of Miniperc**	**Inclusion criteria**	**Surgery**	**Number**	**Sex**		**Side**		**Age mean ± SD (years)**	**Stone Size** **mean ± SD (mm)**	**Follow-up imaging and duration (month)**	**Study quality**
							**M**	**F**	**L**	**R**				
Dundar et al. (15)	CCT	3b	12–20 F	≤ 2 cm, any location	Microperc	16	8	8	8	8	7.9 ± 3.6	12.1 ± 4.3	KUB/USG, 1–2 day	7
					Miniperc	27	13	14	14	13	9.5 ± 5.4	13.4 ± 4.8		
Karakan et al. (13)	CCT	3b	14 F	≤ 2.5 cm, any location	Microperc	42	24	18	22	20	40 ± 13.2	17 ± 3.2	CT,1	7
					Miniperc	32	18	14	16	16	42 ± 14.1	16.4 ± 3.7		
Karatag et al. (17)	CCT	3b	18 or 20 F	1–2 cm, any location	Microperc	56	31	25	N	N	7.63 ± 5.04	13.4 ± 3.4	KUB/USG,1	6
					Miniperc	63	26	37	N	N	9.32 ± 4.98	14.8 ± 3.7		
Tok et al. (14)	CCT	3b	12–20 F	1–2 cm, lower pole	Microperc	58	34	24	34	24	45.90 ± 14.44	13.97 ± 3.62	KUB/USG, 1	7
					Miniperc	40	24	16	22	18	43.08 ± 12.31	16.13 ± 6.97		
Kiremit et al. (16)	CCT	3b	20 F	1–2 cm, any location	Microperc	89	46	43	40	49	40.1 ± 20.32	13.4 ± 2.	KUB/USG, 1	7
					Miniperc	110	56	54	56	54	25.05 ± 20.75	16.8 ± 3.3		
Zhang et al. (18)	CCT	3b	16–18 F	Any location	Microperc	30	22	8	N	N	1.3 (0.8–2.2)	N	CT, 1	6
					Miniperc	43	31	12	N	N	4 (3–8)	N		

*Miniperc, mini-percutaneous nephrolithotomy; Microperc, micro-percutaneous nephrolithotomy; CCT, case control trials; N, Not available; M, Male; F, Female; L, Left; R, Right; KUB, X-ray of kidney, ureter, and bladder; CT, computerized tomography; USG, ultrasonography*.

### Stone-Free Rate

**Figure 2:**
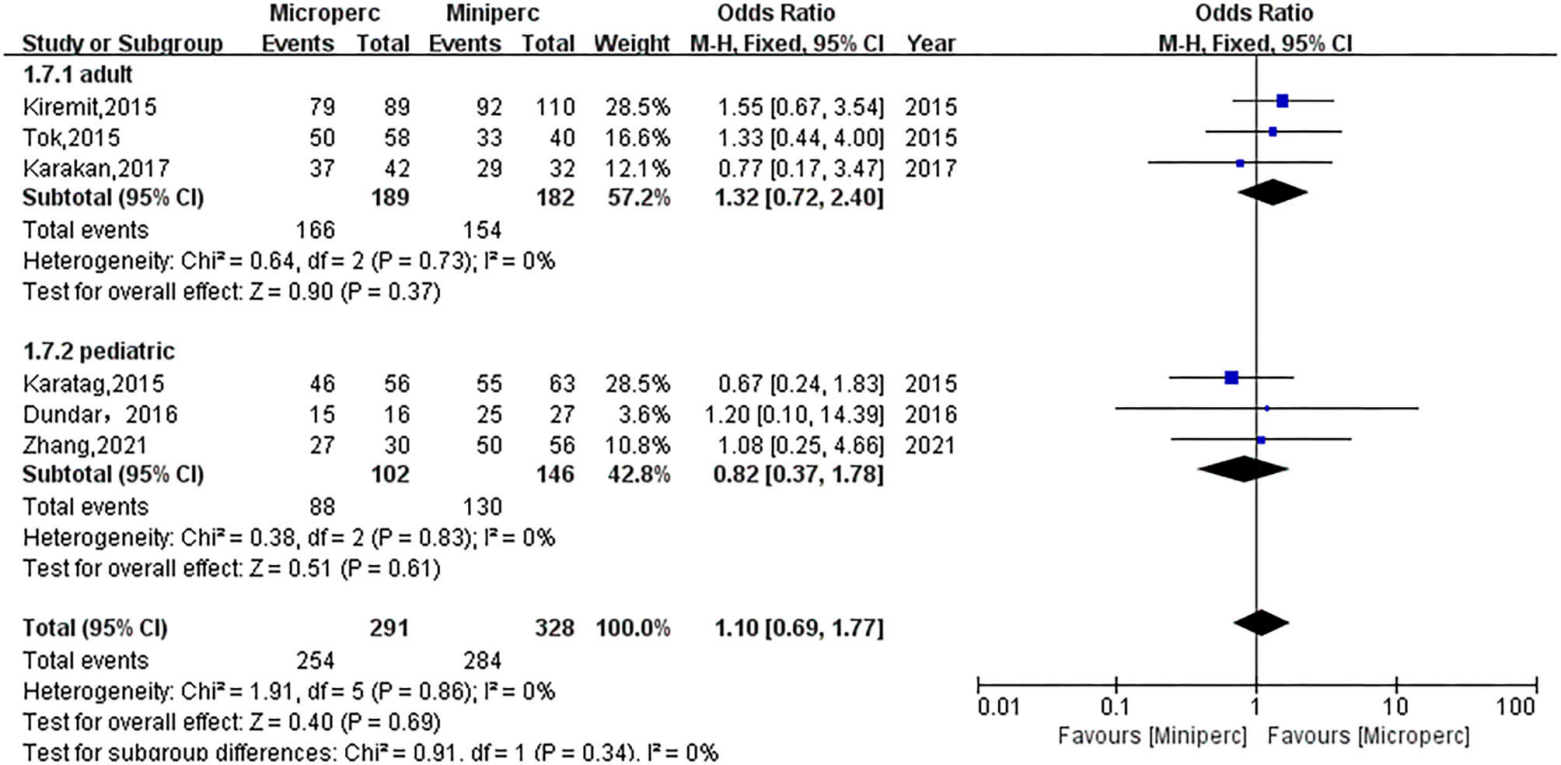
Forest plot for SFR.

All the six studies compared the SFR of Microperc group and Miniperc group (13–18). The stone-free criterion was defined as either no residual stone or fragment with diameter <4 mm. Reexamination was done by performing computed tomography (CT), kidney-ureter-bladder, or ultrasound within 1 month after the operation. In Microperc group, the overall SFR was 87.29% (254/291), while the SFR of Miniperc group was 86.59% (284/328). As the heterogeneity was low among these studies (*P* = 0.86, *I*^2^ = 0%), fixed-effect model indicated that Microperc and Miniperc were statistically similar with respect to SFR (OR: 1.11; 95% CI: 0.69, 1.77; *P* = 0.69; Figure 2). In subgroup analyses, we divided the meta-analysis into adult kidney stones and pediatric kidney stones. The SFRs of Microperc and Miniperc were similar for pediatric kidney stones (OR: 0.82; 95% CI: 0.37, 1.78; *P* = 0.61; Figure 2), while also for adult kidney stones there were no significant differences (OR: 1.32; 95% CI: 0.72, 2.40; *P* = 0.37; Figure 2).

### Operative Time

**Figure 3:**
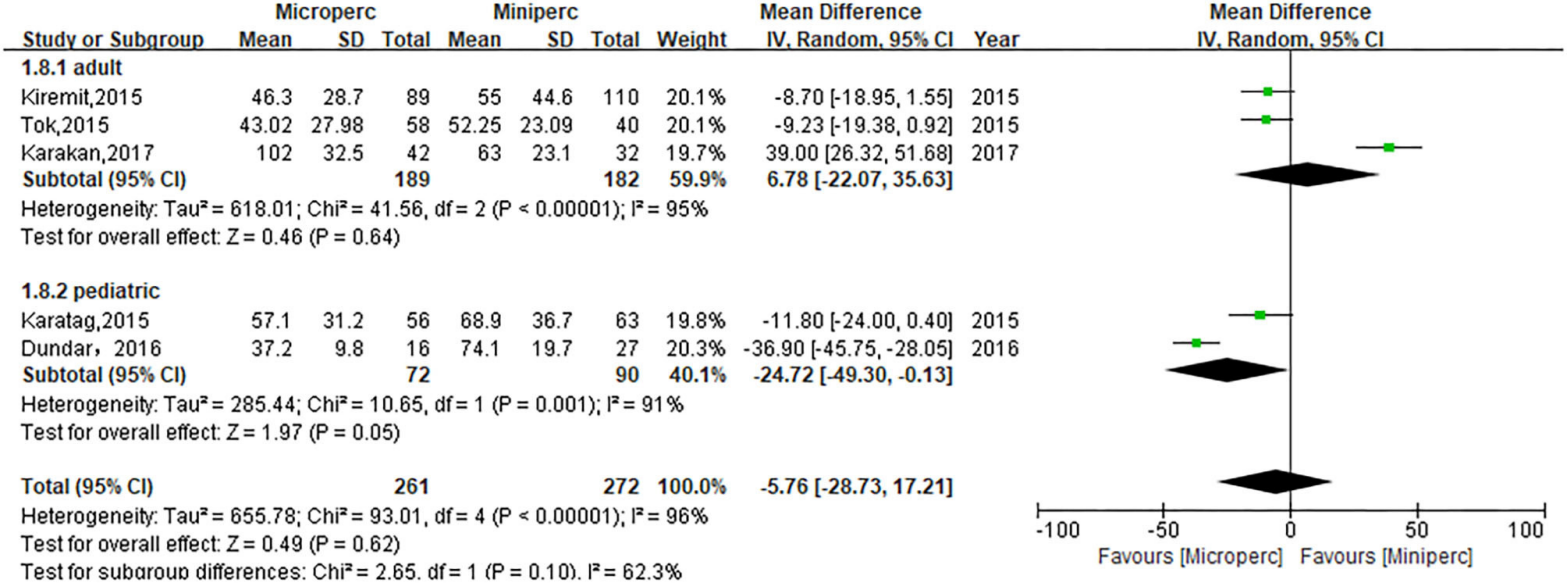
Forest plot for operation time.

The operative time of Microperc vs. Miniperc for renal stones was assessed based on five studies (13–17). The pooled result showed that the heterogeneity was high among these studies (*P* < 0.00001, *I*^2^ = 96%), and the random-effect model indicated that the two techniques were statistically similar with respect to operative time (WMD: −5.76; 95% CI: −28.73, 17.21; *P* = 0.62; Figure 3). In subgroup analyses, our pooled results showed that the operative time of Microperc was shorter for pediatric kidney stones (WMD: −24.72; 95% CI: −49.30, −0.13; *P* = 0.05; Figure 3), while for adult kidney stones, Microperc and Miniperc had no significant differences (WMD: 6.78; 95% CI: −22.07, 35.63; *P* = 0.64; Figure 3).

### Hospital Stay Time

**Figure 4:**
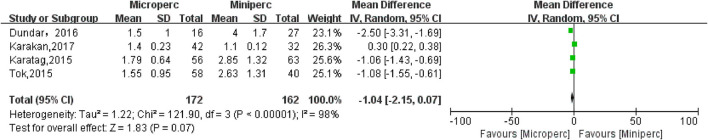
Forest plot for hospital stay time.

The hospital stay time for Microperc vs. Miniperc was measured based on four studies (13–15, 17). As the heterogeneity was high (*P* < 0.00001, *I*^2^ = 98%), the random-effect model was used. Results found that the hospital stay time was shorter in the Microperc group (WMD: −1.04; 95% CI: −2.15, 0.07; *P* = 0.07) (Figure 4), but the differences between the two procedures were not statistically significant.

### Hemoglobin Drop Level

**Figure 5:**
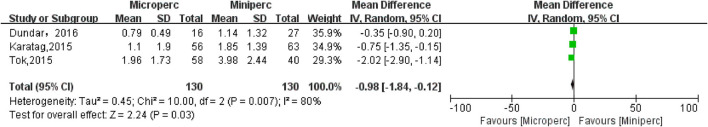
Forest plot for hemoglobin drop.

Hemoglobin drop level in Microperc vs. Miniperc for renal stones was measured based on three studies (14, 15, 17). The heterogeneity was high (*P* = 0.007, *I*^2^ = 80%), and the random-effect model indicated that Miniperc was associated with a larger hemoglobin drop (WMD: −0.98; 95% CI: −1.84, −0.12; *P* = 0.03) (Figure 5).

### Renal Colic Requiring D-J Stent Insertion

**Figure 6:**
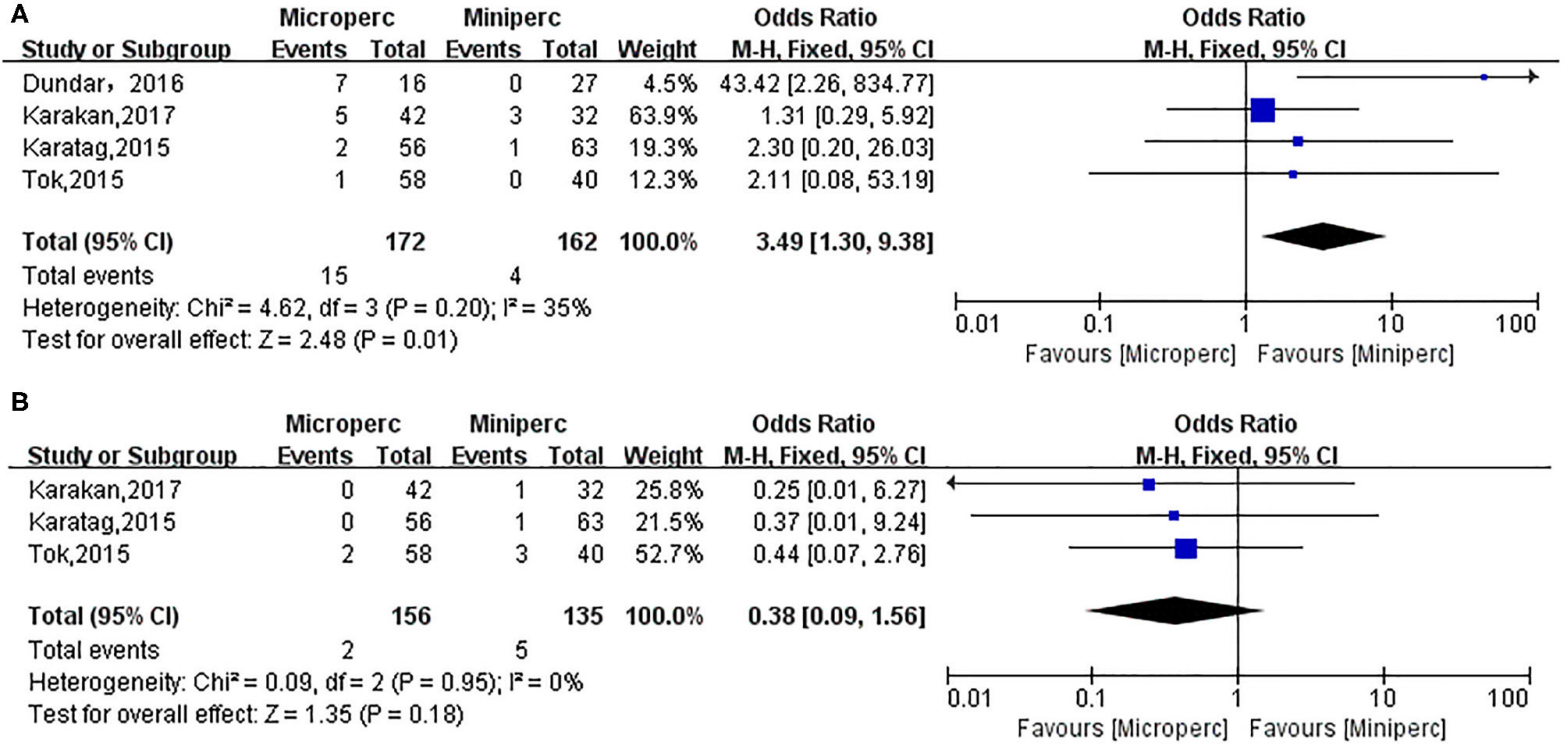
Forest plot for renal colic requiring D-J stent insertion **(A)** and urinary tract infection **(B)**.

Some patients required D-J stent insertion because of larger fragments in the ureter causing renal colic. The heterogeneity was low among four included studies (*P* = 0.20, *I*^2^ = 35%) (13–15, 17), and the fixed-effect model indicated that the requirement for D-J stent insertion in Microperc group was higher than in Miniperc group (OR: 3.49; 95% CI: 1.30, 9.38; *P* = 0.01; Figure 6A).

### Urinary Tract Infection

Urinary tract infection was assessed based on three studies (13, 14, 17). Urinary tract infection was defined as positive urine culture and treatment with appropriate antibiotics. The heterogeneity was low among these studies (*P* = 0.95, *I*^2^ = 0%), and the fixed-effect model indicated that the two techniques were statistically similar with respect to urinary infection (OR: 0.38; 95% CI: 0.09, 1.56; *P* = 0.18; Figure 6B).

### Sensitivity Analysis and Publication Bias

We removed individual studies and found no source of heterogeneity. A funnel plot was used to assess the potential publication bias, and there was no publication bias in this meta-analysis (Supplementary Figure 1).

## Discussion

PCNL has been widely used for the treatment of renal stones since 1970 (19). Its high SFR is accompanied by greater number of complications, such as blood loss and pain, which can be reduced by reducing the diameter of the percutaneous tract (20–22). Miniperc and Microperc have both been used equally effectively for treating kidney stones. Our meta-analysis showed that Microperc could produce an SFR comparable with that of Miniperc, but the complications such as hemoglobin drop and urinary infection associated with Microperc were lower. Besides, we found that Microperc needs shorter hospital stay time and operative time compared to Miniperc, although this difference was not statistically significant. The advantage of Miniperc over Microperc is that a flexible or rigid nephroscope can be moved through the sheath, and lithotripsy can be continued when fragments move into other calyces (21). To our knowledge, this is the first meta-analysis comparing the safety and effectiveness of Microperc vs. Miniperc.

Miniperc was initially used in 1997 for treating pediatric urinary stones (23). Jackman et al. (24) later developed it for treatment in adults. Miniperc usually refers to a percutaneous nephroscope with a nephrostomy tract <20 F (5). Previous studies have confirmed that complications of PCNL could be reduced by reducing the diameter of the percutaneous tract (25). Miniperc can produce an SFR comparable with that of standard PCNL, but complications such as blood loss and pain are reduced (21, 22). Retrograde intrarenal surgery (RIRS) is another procedure for the treatment of upper urinary calculi. A high-quality meta-analysis showed that Miniperc provided significantly higher SFR compared with that of RIRS; however, Miniperc was accompanied with a higher incidence of post-operative complications (26).

In 2011, Desai et al. (27) first used Microperc to fragment stones. The tract of Microperc is smaller than that of Miniperc and standard PCNL, and the puncture and lithotripsy could be completed in one step (27). The see-through needle helps the surgeon puncture into the desired calyx. Microperc is widely used for medium-sized renal stones, and can produce an SFR of 93% (28). For lower-pole stones, the SFR of Microperc can also reach 85.7% (29). Microperc can produce an SFR as high as that of Miniperc; however, Microperc is accompanied with lower blood loss and hospitalization time (14). Compared with RIRS, Microperc produced a significantly higher SFR; however, it is accompanied by a more significant drop in hemoglobin and a more extended hospital stay (30). For pediatric renal stones, the SFR of Microperc was 93.8%, hemoglobin drop was 0.79 ± 0.49 g/dL, and no patient required blood transfusion (15). This indicated that Microperc can be used to treat children with kidney stones. The disadvantage of Microperc is the need to pay attention to the large intrapelvic pressure. Moreover, Microperc cannot exclude fragments, so it is necessary to ensure that the stones are dusted rather than fragmented (21).

SFR is a key indicator to evaluate the effectiveness of lithotripsy. Different studies have different standards for the definition of clear stone. The imaging follow-up methods are different after surgery. Four studies were followed by kidney-ureter-bladder or ultrasound (14–17), and two studies were followed by reexamination by CT (13, 18). The review time was 1 month after surgery, and one study conducted a review 48 h after surgery (15). The standard for SFR was no residual stone or asymptomatic fragments of size < 4 mm. Our pooled results showed that Microperc and Miniperc were statistically similar with respect to SFR. This result indicated that the SFR of Microperc does not reduce when the tract size is reduced. In subgroup analyses, we divided the meta-analysis into adult kidney stones and pediatric kidney stones. The SFR of Microperc was similar to that of Miniperc in both adult and pediatric groups. This demonstrated that there was no difference between the two surgical methods in the treatment of stones in children and adults. We found that the overall SFR of Microperc is 87.29% (254/291), and the SFR of Miniperc is 86.59% (284/328). These results show that both Microperc and Miniperc are very effective in treating moderately sized kidney stones. The SFRs of Miniperc and Microperc were also similar for lower pole stones (14).

Five studies showed that Microperc was accompanied with a shorter operative time (14–18), but one study demonstrated the opposite result (13). Our pooled result showed that Microperc and Miniperc were statistically similar with respect to operative time. In subgroup analyses, our pooled results also showed that Microperc was accompanied by a shorter operative time for pediatric kidney stones. This shows that Microperc is more efficient, especially for treating pediatric stones. Different definitions of operative time resulted in greater heterogeneity among the studies. The variations among studies during the surgery, such as energy source (laser or ultrasonic lithotripsy), diameter of the laser fiber, the optics (flexible), irrigation through the pump, ureteral double J stent inserted, and nephrostomy catheter placement, also caused the differences in operation time in the studies (21).

Complications are important indicators to evaluate the safety of a surgery. Our pooled results indicated that the two techniques were statistically similar with respect to urinary infection (OR: 0.38; *P* = 0.18). However, we found that D-J stent insertion ratio was higher in Microperc (OR: 3.49; *P* = 0.01). It can be explained that Microperc is done to dust the stone and leave the fragments to be spontaneously expelled, while stones and fragments are generally removed in Miniperc. So, Microperc needs more D-J stent insertion. The implanted double J tube was removed after the reexamination found that the residual stone was completely removed without any subsequent intervention.

The tract size of PCNL was significantly associated with blood loss (25). Our pooled results showed that hemoglobin drop was larger in the Miniperc group (WMD: −0.98; *P* = 0.03). The reason could be that the enlargement of the nephrostomy tract increases the damage to the renal parenchyma and renal vascular system (22). In the four studies that we included, all patients in the Microperc group did not require blood transfusions. However, in the Miniperc group, Karatag et al. found that 7.9% (5/63) of the patients required blood transfusions (17). According to the calculations by Dundar et al. (15), 7.4% (2/27) of the patients required blood transfusions.

We demonstrated that the hospital stay time for Microperc was shorter, although not statistically significant. The possible reason was that Microperc involved less damage and less post-operative discomfort (26). Besides, Microperc was associated with a lower rate of urinary tract infection and hemoglobin drop. Furthermore, Microperc was more likely not to use percutaneous nephrostomy tube.

There are several limitations in our meta-analysis. Most importantly, only six retrospective case-control trials were included and analyzed, and the quality of the literature was relatively low. In addition, the heterogeneity was high among the important indicators such as operation time, hospital stay time, and hemoglobin drop. Although the random-effect model was used, the results may be biased. Besides, the definition of SFR and the follow-up time were different in different studies. Moreover, many studies did not list out the specific complications. Finally, the limited studies that were included and the limited number of patients involved in the study could lead to reduced confidence in the results.

## Conclusions

Our meta-analysis demonstrated that Microperc could produce an SFR comparable with that of Miniperc. Microperc was associated with lower hemoglobin drop, but Miniperc was associated with lower renal colic rates. In addition, the operation time and hospital stay time for these two procedures were similar.

## Data Availability Statement

The original contributions presented in the study are included in the article/Supplementary Material, further inquiries can be directed to the corresponding author/s.

## Author Contributions

XG wrote the manuscript. LP collected and analyzed the data. WW analyzed the data. KX helped review and revise the manuscript. TJ helped design the study and revise the article. All the authors have read and approved the manuscript.

## Conflict of Interest

The authors declare that the research was conducted in the absence of any commercial or financial relationships that could be construed as a potential conflict of interest.

## Publisher's Note

All claims expressed in this article are solely those of the authors and do not necessarily represent those of their affiliated organizations, or those of the publisher, the editors and the reviewers. Any product that may be evaluated in this article, or claim that may be made by its manufacturer, is not guaranteed or endorsed by the publisher.

## Supplementary Material

Supplementary Figure 1 Funnel plot evaluating the publication bias.
