# Supplementary figures and images for: Comparison of Micro-Percutaneous and Mini-Percutaneous Nephrolithotomy in the Treatment of Renal Stones: A Systematic Review and Meta-Analysis
Source: Front Surg. 2021 Oct 4;8:743017. doi: 10.3389/fsurg.2021.743017 (PMC8522995; doi:10.3389/fsurg.2021.743017)

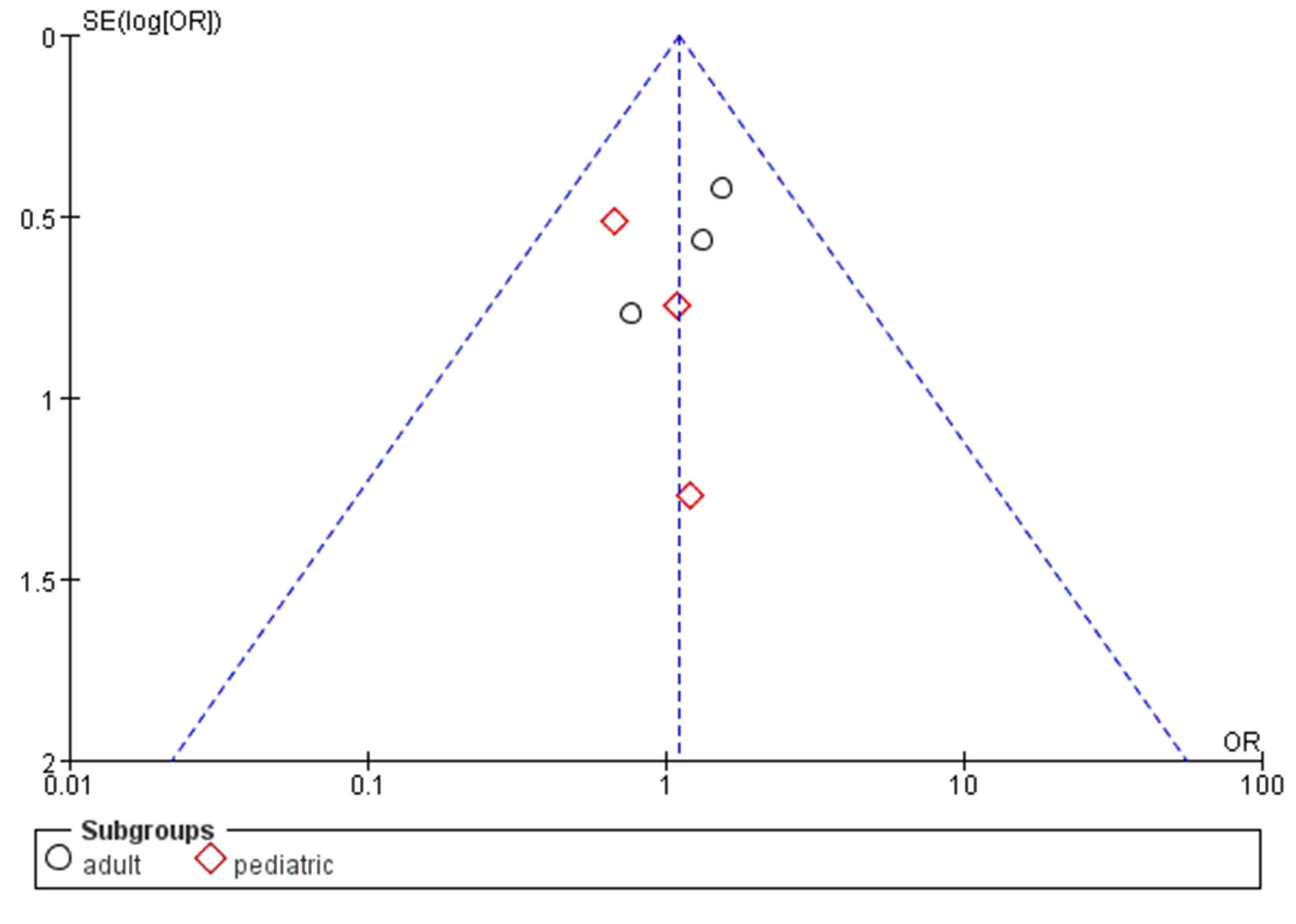

Supplement: Supplementary Figure 1 — Funnel plot evaluating the publication bias. [file Image_1.PNG]
